# The Immune System in Cancer Pathogenesis: Potential Therapeutic Approaches

**DOI:** 10.1155/2016/4273943

**Published:** 2016-12-26

**Authors:** Pankita H. Pandya, Mary E. Murray, Karen E. Pollok, Jamie L. Renbarger

**Affiliations:** ^1^Indiana University School of Medicine, Riley CCRC 2641, 705 Riley Hospital Drive, Indianapolis, IN 46202, USA; ^2^Indiana University School of Medicine, Riley CCRC 2623, 705 Riley Hospital Drive, Indianapolis, IN 46202, USA; ^3^Indiana University School of Medicine, Research Building R4, 302D1044 West Walnut St., Indianapolis, IN 46202, USA; ^4^Indiana University School of Medicine, Riley CCRC 2616, 705 Riley Hospital Drive, Indianapolis, IN 46202, USA

## Abstract

Interplay among immune activation and cancer pathogenesis provides the framework for a novel subspecialty known as immunooncology. In the rapidly evolving field of immunooncology, understanding the tumor-specific immune response enhances understanding of cancer resistance. This review highlights the fundamentals of incorporating precision medicine to discover new immune biomarkers and predictive signatures. Using a personalized approach may have a significant, positive impact on the use of oncolytics to better guide safer and more effective therapies.

## 1. Introduction

The link between the immune system and cancer has been widely appreciated for over a century and was first highlighted by Rudolph Virchow over 150 years ago [1]. The underlying basis for this relationship between cancer and immunity involves three basic principles of how the immune system acts to defend and protect an individual: it detects “nonself” antigens from pathogens or infected/malignant cells; it encompasses effector functions to specifically target and destroy the pathogen or infected/malignant cells while protecting the host; and it develops immunological memory via the adaptive immune responses for subsequent defense mechanisms following an injury or an attack against the host [2]. Through this process, the immune system has acquired characteristics that give rise to the paradigm known as immunoediting, which provides a balance between immune surveillance and cancer progression in the realm of oncology [3, 4]. This multifaceted mechanism consists of the three primary phases: elimination, equilibrium, and escape, that contribute to cancer elimination, dormancy, and progression, respectively [4]. Interestingly, this ability of cancers to evade or escape the immune response is now recognized to be one of the most distinguished cancer hallmarks, which provides the platform for treatments within the context of immunotherapies. Although the initial utilization of immunotherapy for cancer treatments dates back to the early nineteenth century, suggestive of work done by William B. Coley and colleagues [1, 5], it was the more recent scientific advances that have helped elucidate innovative approaches for implementing immunotherapies to eradicate and/or treat various cancers. These advances have made the concept of immunooncology and cancer immunotherapy more clinically relevant. This review highlights the emerging and evolving findings that contribute to the understanding of immunooncology, as well as emphasizing the importance of relevant immunotherapies for potential therapeutic interventions in cancer treatments.

## 2. Cancer Biology

Being ranked as the second major cause of death in the United States, incidence of cancer and cancer-related mortality rates have been on the rise [6]. Onset of cancer stems from several types of spontaneous and induced genetic mutagenesis, some of which include altered glycosylation patterns, gain or loss of chromosomes, and translocation [7]. Altered glycosylation patterns contribute to cancer heterogeneity by regulating growth of cancer cells via glycosylation of certain growth factor receptors [7]. These mutations occur in specific types of genes known as oncogenes or tumor suppressor genes which are known to promote or inhibit cell growth, respectively [7]. Interestingly, epigenetic alterations such as DNA methylation also play a role in cancer pathogenesis [8]. For example, hypermethylation is known as a mechanism for silencing tumor suppressor genes [9, 10], whereas, DNA hypomethylation of mobile DNAs causes gene activation and is observed to occur in several forms of cancers [9, 10]. These epigenetic mutations can affect a broad range of tissues or organs resulting in distinct cancer types such as prostate cancer and breast cancer [10]. While the onset of cancer can be initiated by hereditary factors, environmental factors such as diet, exposure to certain chemicals (carcinogens) or radiation, and lifestyle choices such as smoking are also contributing factors. Although the underlying cause for many of these cancers can be attributed to inherited or acquired genetic mutations, cancer pathogenesis greatly varies depending on the tissue or organ that is affected, the molecular/genetic mechanisms involved, and the treatment options available for that particular type of cancer.

The terminology used to describe the different forms of cancer results in another layer of complexity to the field of oncology. For instance the cancers that remain localized to their site of origin are referred to as “primary cancer,” while secondary or metastatic cancers are those that migrate to other locations of the body [11]. It is important to consider that the terms “cancer” and “tumor” cannot be substituted for one another [11]. Tumor refers to the mass of abnormal cell growth (neoplasms) which can be benign or malignant (injurious) [11]. These benign tumors remain localized at their site of origin, whereas malignant tumors are referred to as being cancerous, and they metastasize to other organs [11]. Notably, while the metastatic cancers still possess the transformed cells from the original primary cancer, they do acquire distinct characteristics over time that help distinguish them from the primary cancer [12]. The mechanism by which metastasis occurs involves certain transformed cells being able to detach themselves from the primary cancer growth (neoplasm) and travel through other sites via lymphatic flow or blood circulation [11]. These metastatic cancer cells secrete enzymes such as matrix metalloproteinases that degrade extracellular matrix proteins and utilize chemotaxis to enable them to migrate to other locations. While not all cancer cells in circulation survive, the ones that do survive can attach themselves to endothelial cell lining of the capillary venules or blood vessels and migrate to the secondary tissue/organ using complex signaling pathways [13]. Subsequently, these cancer cells can proliferate via mechanisms that induce angiogenesis [14]. Growth of metastatic cancer depends on getting adequate blood supply for nutrients and oxygenation and being able to efficiently remove cellular waste through a process known as angiogenesis [14, 15]. The hypoxic environment of cancers induces expression of proteins such as hypoxia-inducible factor-1alpha (HIF-1*α*) that can regulate expression of angiogenic growth factors like vascular endothelial growth factor (VEGF) [14, 15], though a wide variety of other proteins such as cytokines, interleukins, and other growth factors also contribute to angiogenesis for cancer proliferation [15].

Similar to the complexity of cancer pathogenesis and its terminology, classification of the types of cancers also remains to be a challenging concept due to various factors involved. Cancer or tumor can originate from several types of tissues such as epithelium, mesenchyme (connective tissue/bones), or glands and depending on their site of origin they are referred to as carcinoma, sarcoma, or adenocarcinoma, respectively [16]. Cancers that occur within the lymphatic system and affect the lymphoid organs are known as lymphomas [17], whereas tumors that originate in the bone marrow such as myeloma or leukemia affect the production of plasma cells in the case of myeloma or affect the production of erythrocytes and leukocytes in leukemia [18]. Interestingly, the pathophysiology of these cancers can greatly vary among adults and children [19]. It has been reported that cancers in children progress faster and tend to be more aggressive compared to those observed in adults [19]. Such differences in cancer pathogenesis further contribute to the variability observed in patient care and treatments.

Due to the complex nature and heterogeneity present in cancer pathogenesis, treatment for any one particular type of cancer is difficult to develop. However, huge strides have been made in developing therapies that can target the six hallmarks of cancer: evading growth suppressors, activating invasion and metastasis, enabling replicative immortality, inducing angiogenesis, resisting cell death, and sustaining proliferative signaling [20]. While recent advances are focused on targeting mutations or symptoms associated with these six cancer hallmarks as potential cancer-specific treatments, the more beneficial route would be to understand and target a common key player that is present in cancer as a whole, such as the immune system. To gain insight into how the immune system can be exploited as a treatment option for cancer, it is critical to understand that key components of the immune system and the role it has in oncogenesis. The succeeding sections of this review highlight these important concepts and shed light on how the interaction between cancer and the immune system resulted in development of the immunooncology paradigm.

## 3. Immune System 

**Figure 1:**
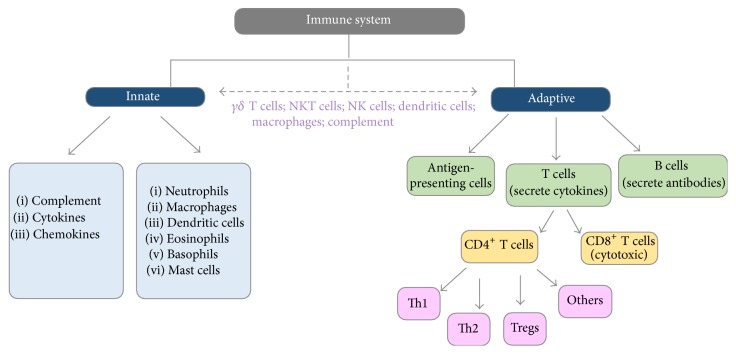
Overview of the immune system: innate and adaptive immunity. An evolutionary bridge between both forms of immunity is observed due to the presence of *γδ* T cells, NKT cells, NK cells, dendritic cells, macrophages, and complement proteins. The innate immune responses include cells and soluble components that are nonspecific, fast-acting, and first responders in inflammation. In contrast, adaptive immunity encompasses immune components that are more specific for targeted antigens and capable of forming immunological memory [25, 27, 38].

**Figure 2:**
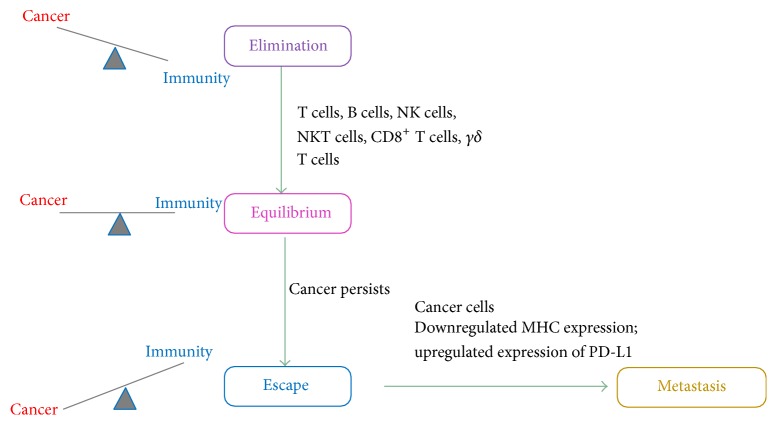
The cancer immunoediting process. There are three phases in the cancer immunoediting process: elimination, equilibrium, and escape [4]. Elimination phase involves effector function of the immune cells to target and eradicate cancer. In the equilibrium phase a balance is obtained between progression of cancer and cancer elimination by the immune system. If the cancer persists then it overwhelms the immunity and escapes to go on and metastasize to the other organs [4, 40, 53].

The immune system is comprised of several types of soluble bioactive molecules, cytokines, proteins, and cells that collectively form the multifaceted network of biochemical processes that recognize and defend against “nonself” proteins or antigens [2]. To provide protection and maintain the host's normal state of homeostasis, the immune system consists of two forms of immune responses: innate and adaptive (Figure 1), [2]. Nonspecific and immediate immune responses are classified as innate due to their fast-acting nonspecific response against foreign antigens such as pathogenic microbes, allergenic antigens, or non-self-proteins or molecules (Figure 1) [2, 21]. While innate immunity is short-lived and not able to form an immunological memory, it is still able to distinguish between “self” and “nonself” or different groups of pathogens via receptors such as toll-like receptors (TLRs) and others that recognize specific danger associated or pathogen associated molecular patterns (DAMPs or PAMPs) [21]. For example, TLR7 is an intracellular receptor that can recognize single stranded RNA but it can also suppress induction of T regulatory cells (Tregs) which is helpful in the tumor environment [1, 22]. Other mechanisms by which the innate immunity imparts immediate protection to the host involve soluble bioactive proteins such as cytokines and complement proteins [2]. Notably, the cytokines have various functions depending on the microenvironment they were secreted in, the cells they were secreted by, the location of the receptor it binds to, and the signaling pathways that are activated following their binding to the receptor [23]. On the other hand, while the complement proteins have three major signaling pathways that they are activated by (classical, alternative, and the lectin pathways), all pathways result in activated complement proteins. Upon activation, complement proteins function in opsonization, act as chemoattractant for other immune cells, and mediate cell/pathogen death by formation of membrane attack complex for lysis [24].

The key players in cell-mediated innate immune responses involve phagocytes and natural killer (NK cells) [25]. These phagocytes (neutrophils, monocytes, and macrophages) facilitate immediate host protection by engulfing cells that express non-self-antigens or altered self-antigens and killing them with lysosomal enzymes (Figure 1) [25]. On the other hand, NK cells confer immune protection using major histocompatibility complex I (MHC class I) proteins which are universally expressed on cell surface of all nucleated cells [25]. These NK cells secrete perforin and granzyme to induce apoptosis of cells that have abnormal or altered MHC class I expression if the cell has been compromised or a pathogen is expressed [25]. Other cells such as eosinophils, basophils, and mast cells that release inflammatory mediators like chemotactic leukotrienes also contribute to the cellular innate immunity by recruiting more immune cells to the inflammation/injured site (Figure 1) [2]. It is important to note here that, in humans, proteins known as human leukocyte antigens (HLA) are the equivalent to the MHC found in most vertebrates. Similar to NK cells, there are also cells known as NKT cells which possess qualities of both the NK and the T cells [25].

Contrary to the innate immune response, the adaptive immunity involves the development of immunological memory due to specific forms of immune responses targeting the antigens (Figure 1) [2]. This form of immunity occurs over time and is not characterized as being a rapid response due to naïve lymphocytes, such as the T and B cells, gaining the ability to differentiate and mature into either effector T cells or antibody-secreting B cells (plasma cells) (Figure 1) [26]. There are two types of T cells present in the immune system that are distinguished by their T cell receptor type: *αβ* T cells and *γδ* T cells (Figure 1) [27]. Only a small subset of T cells are classified as *γδ* T cells and can recognize “nonself” molecules by pattern recognition, thereby not requiring MHC-mediated antigen presentation (Figure 1) [27]. On the other hand, *αβ* T cells are further broken down into two other subsets known as CD4^+^ T cells and CD8^+^ T cells (Figure 1) [28, 29]. Maturation of naïve CD4^+^ T cells to effector CD4^+^ T cells involves costimulation between major histocompatibility complex II (MHC class II) which are only present on antigen-presenting cells (B cells, macrophages, and dendritic cells) and T cell receptor on the naïve CD4^+^ T cells (Figure 1) [28, 29]. Depending on the cytokine milieu present in the microenvironment and the presence of certain transcription factors during the time at which the costimulatory signal occurs, the CD4^+^ T cells can differentiate into several subsets of effector T cells such as T helper 1 (Th1) cells, T helper 2 (Th2) cells, or Tregs (Figure 1) [29]. Each of these subsets of CD4^+^ T effector cells can produce and secrete certain cytokines that modulate immune response accordingly [29]. For instance, Th1 cells produce IFN-*γ* and interleukin-2 (IL-2) and play a role in autoimmunity. Notably, Th2 cells produce interleukins 4, 5, 10, 13, and 31 (IL-4, IL-5, IL-10, IL-13, and IL-31) and regulate immune responses involving extracellular pathogens as well as allergic diseases [29]. Tregs, on the other hand, help reduce inflammation via production of transforming growth factor-beta (TGF-*β*), interleukin-35 (IL-35), and IL-10 [29]. Similar to NK cells in innate immunity, naïve CD8^+^ T cells rely on MHC class I for maturation into effector cytotoxic T cells [28]. MHC class I molecules present on nucleated cells can recognize and bind to peptides derived from nonself antigens and altered or abnormal self-antigens [2, 28]. CD8^+^ T cells via the specific T cell receptor bind to the antigen/MHC class I complexes on the antigen-presenting cells (i.e., target cells) resulting in release of perforin and granzymes from CD8^+^ T cells and death of the target cell [28]. Both types of T cells (CD4^+^ and CD8^+^) express other cell surface markers such as CD28 and CTLA-4 that participate in activating or inhibiting the naïve T cells, respectively, by binding to CD80/CD86 on antigen-presenting cells during costimulatory signaling [30]. Interestingly, the cell surface marker PD-1 on T cells which binds to PD-L1 and PD-L2 on antigen-presenting cells also inhibits T cell activation [30]. Notably, certain cancer cells can also induce PD-L1 expression as mechanism to suppress and evade the immune system [30].

For maturation and activation of B cells, antibody-secreting effector functions can be activated by T helper cell-dependent and cell-independent mechanisms [31] resulting in a wide range of antibodies which are specific for the type of immune response that is initiated [31]. Antibodies are also commonly referred to as immunoglobulins (Ig). They all possess a fragment antibody binding (Fab) domain that can bind to numerous antigens and a fragment crystallizable (Fc) domain that can bind to its corresponding Fc receptors on effector cells to mediate effector functions such as antibody-dependent complement cytotoxicity (ADCC) [32]. Whereas all naïve B cells express membrane-bound IgD and IgM [32], various other antibody isotypes such as IgA, IgG, and IgE are also produced by immediate and long-term memory plasma cells through immunoglobulin class switching, affinity maturation, and somatic hypermutations [32]. Among these isotypes, certain antibodies such as IgA and IgG also have different subsets that they can be classified as isotypes [32]. The various antibody isotypes and subsets can have distinct functions that they carry out in different conditions [32]. However, in general, antibodies function to neutralize the antigen by binding to it, initiating ADCC, interacting with components of the classical complement pathway to induce complement dependent cytotoxicity (CDC), and also binding to antibody receptors on specific cells to activate their effector functions [32].

Similar to NK cells present in innate immunity, the adaptive immune responses also have their own version of NK cells known as NKT cells. These cells possess qualities of both the NK and the T cells, because they express NK 1.1, a natural killer cell-associated surface marker, along with the expression of T cell receptors (TCRs) [33]. However, some of their TCRs can differ from the normal TCR, thus classifying them as invariant NKT cells [33]. These NKT cells are able to identify and bind to self or nonself lipids/glycolipids through the expression of CD1d molecule on antigen-presenting cells after which they secrete several cytokines such as IL-12 and IFN-*γ* for activation of other immune response [33].

While innate and adaptive immune responses have distinct characteristics that distinguish them from one another, both forms of immunity work in tandem [34]. The innate immune response is initiated as the first line of defense prior to adaptive immunity [34]. However, if the innate immune responses become overwhelmed by the antigens and are not sufficient for their clearance, then the onset of adaptive immunity occurs to aid in antigen removal and form immunological memory for subsequent exposures [34]. Some of the components that serve as linker between innate and adaptive immunity include dendritic cells, macrophages, and complement protein. Dendritic cell and macrophages can both function in innate immunity by phagocytosing pathogens or innate immunity, but they also contribute to adaptive immune responses by serving as antigen-presenting cells for T cell activation [34]. The role of complement proteins in innate immunity reflects its ability to eradicate cancer cells by formation of the membrane attack complex or by CDC [35]. Moreover, in adaptive immune responses, the complement system can contribute to B cell activation by lowering the activation threshold and by mediating T cell activation and differentiation [35, 36]. Other cells like NK cells, NKT cells, *γδ* T cells, macrophages, dendritic cells, and complement proteins serve as evidence for the evolutionary bridge between innate and adaptive immunity (Figure 1) [25, 27, 37].

## 4. Innate Immunity and Cancer

During cancer pathogenesis several components of the innate immunity are activated in efforts to minimize cancer-mediated inflammation [39, 40]. This process also initiates adaptive immune responses for targeting the cancer via more specific immune mechanisms [39, 40]. Several studies suggest the role for genetic and epigenetic modifications in cancer cells [7]. Notably, these alterations in the cancer cells correlate with the changes observed in the composition of their cell surface proteins, resulting in expression of tumor-associated antigens which can be recognized by complement proteins [41] and thereby predisposing the cancer cells to complement-mediated death [41]. Activation of complement proteins has been reported in local and/or systemic biological fluids of cancer patients, as well as in cancer tissues from patients diagnosed with neuroblastoma, lung cancer, ovarian cancer, and a variety of others [41]. While complement activation promotes mechanisms that aid in eradicating cancer cells, the presence of soluble and membrane-bound complement regulatory proteins (CRPs) that inhibit various steps in the multiple complement signaling pathways help protect cancer cells against complement-mediated injury [41]. Inhibition of the complement cascade also hinders some of the effects of adaptive immune responses because complement proteins have also been reported to play a role in B- and T cell activation/survival [36]. Therefore, CRP-mediated complement inhibition may also result in insufficient activation and expansion of B and T cells that can specifically target the cancer cells [36].

As previously mentioned, genetic and epigenetic modifications result in modified cell surface markers and patterns on the surface of the cancer cell [7]. Notably, one such cell surface marker is MHC class I whose expression becomes altered or reduced in cancer cells [37]. This aberrant or reduced MHC class I expression leads to activation of NK cells via activating receptors present on NK cell surface such as NKG2D which bind to surface glycoproteins known as MICA/B that may be present on the tumors [37]. NK-induced programmed cell death (apoptosis) can occur by several mechanisms such as tumor-necrosis factor-alpha- (TNF-*α*-) dependent release of cytoplasmic granules (perforin and granzymes) that form pores in cell membranes; by antibody-dependent complement cytotoxicity due to the presence antibody receptor (CD16) on NK cell surface; and by the release of cytokines such as IFN-*γ* which mediates activation and maturation of antigen-presenting cells such as dendritic cells [37]. Cells are resistant to NK-mediated lysis due to normal expression of MHC class I that activates inhibitor receptors on NK cells which prevents NK cell induced apoptosis [37].

Other mechanisms by which the innate immunity contributes to cancer pathogenesis include neutrophils which have been more widely known to promote cancer progression [42]. Proteases such as neutrophil elastase present in neutrophil granules facilitate growth of cancer cells [42]. Other proteases in the neutrophil granules assist in cleaving extracellular matrix proteins, thus allowing cancer invasion and metastasis [42]. As previously mentioned, these neutrophils also contain phagolysosomes which contain enzymes like NADPH oxidase which oxidizes superoxide radicals and other reactive oxygen species (ROS). It is well documented that ROS have been reported to not only promote cancer by genetic modifications via DNA damage, but also initiate cytotoxicity through disruption of the cell membrane on the tumors [42].

Various cell types also serve as evolutionary linkers between the innate and adaptive immunity. These cells include dendritic cells, *γδ* T cells, macrophages, and NKT cells [33, 37, 43, 44]. For instance, dendritic cells and macrophages, which function as phagocytes in innate immune responses, can also function as antigen-presenting cells for adaptive immunity [34, 43]. Tumor cells can produce thymic stromal lymphopoietin which promotes upregulation of OX40 ligand expression on dendritic cells and other types of APCs [43]. Expression of OX40 is induced on activated T cells and acts as a secondary costimulatory signal to CD28 signaling on T cells [45]. As a consequence of OX40 ligand and OX40 expression, direct T cell-APC interactions promote T cell activation and subsequent differentiation into the Th2 T cell subset [45]. Additionally, some types of tumor cells have been known to prevent the antigen-presenting capabilities of dendritic cells, thereby inhibiting dendritic cell-dependent T cell activation [43]. One common mechanism by which NKT cells contribute to immune responses to tumor cells is through IFN-*γ* secretion which activates the effector functions of cells such as NK cells or CD8^+^ T cells to mediate tumor lysis via granzymes or perforin [33]. Interestingly, NKT cells' interaction with dendritic cells via CD40 ligand-CD40 signaling, respectively, enables activation and secretion of IL-12 [33]. CD40 is expressed on several types of antigen-presenting cells and acts as a costimulatory signal when bound to CD40 ligand on NK cells, mast cells, macrophages, B cells, epithelial cells, endothelial cells, and activated T cells [33, 45]. In addition, IL-12 can activate NK or CD8^+^ T cells for tumor lysis and suppression of cancer progression [33]. Similar to NK and CD8^+^ T cells, the *γδ* T cells express NKG2D which interacts with MICA/B on tumor cells and promotes secretion of perforin proteins and subsequent tumor lysis [44]. Other mechanisms implemented by the *γδ* T cells for regulating cancer progression involve secretion of IFN-*γ* that can activate NK or CD8^+^ T cells for tumor lysis, recognition of tumor-associated antigens by CD16 (Fc receptor) to mediate antibody-dependent complement cytotoxicity, and the ability of *γδ* T cell receptors to bind to self-antigens such as heat shock proteins that are upregulated in the cancer microenvironment [44].

Though the innate immunity plays a critical part in regulating cancer pathogenesis, another equally important aspect in cancer biology is the role of adaptive immunity [3, 40]. Effector functions of adaptive immunity result in either tumor eradication or proliferation depending on the environmental signals [3, 40]. The next section will highlight the mechanisms by which lymphocytes and APCs promote tumor progression or regression.

## 5. Adaptive Immunity and Cancer

Similar to innate immunity, the adaptive immunity is comprised of several components that can either eradicate cancer cells or promote their proliferation [39]. This form of immune response is capable of targeting antigens specific to the cancer cells by exploiting the effector functions of antibodies, T cells, B cells, and antigen-presenting cells [38]. The central dogma behind the cancer immunity concept involves the formation of neoantigens, such as the new antigens that are formed due to tumorigenesis/oncogenesis, which are phagocytosed by antigen-presenting cells (APCs) or pinocytosed by dendritic cells for antigen processing [39]. MHC class II molecules present exogenous peptides of tumor antigens, whereas MHC class I molecules present endogenous peptides derived from cancer antigens [38]. The processed tumor-associated antigens are then presented by MHC class II and MHC class I molecules on the APC to the antigen-specific T cell receptor on CD4^+^ T cells or CD8^+^ T cells, respectively [39]. Activation of CD4^+^ T cells by MHC class II on APC primes them for subsequent exposures to that particular antigenic peptide/MHC class II complex, thus forming memory T cells [39, 46]. IL-2 is also produced when T cells are activated and further promotes T cell proliferation [47]. The cytokine milieu present within the tumor environment at the time of CD4^+^ T cell activation dictates the T cell differentiation pathway as previously mentioned [39]. While it is known that B cells can act as APCs to naïve T cells, activated CD4^+^ T cells (also known as helper T cells) can also interact with naïve B cells to promote their activation [31]. This process is known as thymus-dependent activation of B cells and encompasses two types of signals between T helper cells and B cells: TCR-MHC class II with tumor antigen and a costimulatory signal between CD40 ligand and CD40 [31]. In the absence of this costimulatory signal, the B cells are not able to be activated or proliferate [31]. B cells can also be activated by thymus-independent (TI) mechanisms which involve antigens with highly repetitive structures [31]. Following TI-independent B cell activation, antibodies are secreted which can bind to the tumor-derived antigen. This can initiate tumor cell lysis via ADCC or CDC and also by [32] binding to Fc receptors on NK cells [25]. Similarly, activation of CD8^+^ T cells occurs by interaction of antigen-specific T cell receptors with MHC class I/tumor antigen complexes leading to induction of cytolytic CD8^+^ T cell-mediated lysis of cancer cells [39].

Within this central dogma of cancer immunity, there are several regulatory factors that act as immune checkpoints in the context of adaptive immune responses to mediate either cancer progression or regression. For instance, during the first encounter with antigen/MHC class II on the antigen-presenting cells, it is critical to have two signals delivered between the APC and T cell: antigen-bound MHC class II interacting with the T cell receptor and costimulatory signals [45]. Some of these costimulatory signals include CD28, ICOS, and CD80 (B7.1)/CD86 (B7.2) [30, 45]. The ICOS ligand on APCs interacts with ICOS receptor on T cells, whereas CD28 on T cells interacts with CD80 (B7.1)/CD86 (B7.2) on APCs for costimulation [30, 45]. If these costimulatory signals are not present when activating the naïve T cells, then they will not differentiate or proliferate [30]. Lack of an appropriate costimulatory signal ultimately results in T cell anergy and a state of immune tolerance to cancer cell-associated antigens; under this scenario, adaptive immunity is shut down and cancer progresses [48]. Similarly, immune tolerance is also initiated by CTLA4 on T cells binding to the CD80/CD86 proteins on APCs [30, 45]. Contrary to the binding of CD28 with these proteins, interaction of CTLA4 on CD4^+^ T cells with CD80/CD86 on APCs results in T cell inhibition and mediates downregulation of immune responses [30, 45]. CTLA4 is also expressed by several cancers/tumors and this mechanism corresponds with immune tolerance as seen in cancer progression [49]. Interestingly, the T cells also have a cell surface receptor molecule known as programed cell death protein 1 (PD-1) which can bind to its ligand, PD-L1, on APCs and mediates immunosuppression [30]. Notably, the PD-1 expression has also been reported in multiple other immune cells such as B cells, NK cells, monocytes, dendritic cells, and Tregs [30]. Similar to expression of CTLA4, this PD-L1 protein is also expressed by various types of cancer cells which may be a mechanism for how the cancer escapes immunity [30, 49]. Immune responses in oncogenic environment can also be suppressed by Tregs [50]. Tumors/cancer cells can secrete chemokines like CCL22 that recruit Tregs to the oncogenic site and help suppress effector functions of other T cells that may be necessary to eradicate cancer cells [50, 51]. Collectively, the role of these innate and adaptive immune responses in oncogenesis serves to be the underlying basis for immune surveillance and cancer immunoediting [4, 40].

## 6. Cancer Immunoediting

The role of the immune system in cancer pathogenesis has been a subject of great interest and debate for many decades due to its ability to mediate protection against cancer and promote cancer progression [4, 40]. The role of immune responses in the context of cancer biology is commonly referred to as cancer immunosurveillance [4, 40]. While Paul Ehrlich is recognized as the scientific pioneer behind the immunosurveillance concept, contradictory reports on this concept based on studies conducted by Burnet and Thomas and Stutman's group brought the concept of immunosurveillance to the forefront in oncology [4, 40]. Due to these and other inconsistent reports from studies highlighting the immunosurveillance mechanisms in cancer, the concept was largely rejected [4]. However, with the scientific advancement in genetically modified animal models, design of studies to investigate immunosurveillance was feasible [4, 40]. Consequently, the role of immunity in cancer was reevaluated once again in the 1990s [4, 40]. Several counterarguments against the cancer immunosurveillance theories were discarded with the exploitation of mice models deficient in adaptive immunity (RAG2 knockout mice) or mice lacking components of interferon-gamma (IFN-*γ*) signaling cascade [4]. Notably, studies from these and other animal models deficient in some form of the immune response were highly indicative of immunity protecting against carcinogenesis and tumor formation [4, 40]. Moreover, immune-mediated protection against cancer is not just limited to animal models, rather it has become increasingly clear that immunosurveillance is clinically observed in humans as well [40]. Interestingly, recent reports suggest that a delicate balance between cancer dormancy and progression exists and this balance is the foundation for the principle in oncology known as immunoediting [52]. Three major phases that comprise the immunoediting process in cancer pathogenesis are elimination, equilibrium, and escape (Figure 2), [4, 40]. These underlying immune responses of immunoediting help shape the immunogenicity of various cancers. The outcome of immunoediting may be attributed to factors which include the temporal or spatial location of the cancer, molecular mechanisms involved in transformation from normal to transformed cells, and the inherent genetic factors of the immune system [52]. The elimination phase of the immunoediting process is a component of the cancer immunosurveillance theory and refers to the ability of the innate and adaptive immune system to recognize and eradicate cancer cells (Figure 2) [4, 40, 53]. Mechanisms by which cancer cell lysis occurs are via secretion of perforin from cytolytic immune cells (i.e., NK cells, NKT cells, *γδ* T cells, and CD8^+^ T cells), ADCC, or CDC [3]. The equilibrium phase focuses on the dynamic state of the cancer cells to negatively regulate the immune system leading to a block in the elimination phase of immunoediting and a transition to the equilibrium phase (Figure 2), [4, 40, 53].

In the equilibrium phase, immune responses are still active against the tumor; immune cells help regulate and control cancer growth or metastasis while keeping it in the latent dormant state. The phase of equilibrium is considered to be the longest phase in the immunoediting process [4, 40, 53]. Despite these control checkpoints that are modulated by the immune system, the heterogeneity and genetic variations in cancer enable them to acquire the ability to become immune-evasive and escape the equilibrium state to expand and become detrimental to the host [4, 40, 53]. This escape phase is mediated through several immunosuppressive mechanisms one of which includes downregulation or aberrant expression of MHC class I on the cancer cell surface protecting it from cytotoxic effector functions of immune cells in the innate and adaptive immunity (Figure 2) [39, 54]. Multiple mechanisms such as suppression of tumor antigen expression, induction of antiapoptotic pathways to prevent cytotoxicity, and cancer-induced immunosuppression aid in the escape of cancer cells from the elimination and equilibrium phases of immunity [4, 40, 53]. Notably, it is this escape of cancer cells from immunity and the mechanisms involved in this escape that has been the driving force of investigations focused on the immune-oncology paradigm. Gaining a detailed understanding of immunoediting process in cancers will be critical for development of immunotherapies for cancer treatments.

## 7. Precision Medicine

Precision medicine, a novel approach for patient-specific therapies, is revolutionizing clinical outcomes and standard of care [55, 56]. Therefore, in efforts to treat various forms of cancers, scientific advances have been made towards developing therapies that exploit the immune system [55]. These specific types of cancer treatments that focus on utilizing innate and adaptive immunity are referred to as cancer immunotherapies [55]. Due to the paradigm shift in health care which focuses on precision medicine, more initiative is directed towards establishing immunotherapy-based personalized treatments towards individual cancer patients. These cancer immunotherapies can be classified into several different categories: vaccines, monoclonal antibodies, recombinant cytokines, small molecules, and autologous T cells [55]. The site, the type, and the stage that the specific cancer is in dictate the type of therapy that is best suited for the patient.

Several FDA approved and clinical trial immunotherapies have been developed to treat various forms of cancer [55]. Despite the initial promising success rates of these therapies, the vast majority of patients relapse [56]. This can be attributed to various factors that distinguish individual patients such as age, gender, chemotherapy regimen, and site/type of cancer, all of which play a functional role in cancer genomics and serve as the fundamentals for precision medicine [56]. These cancers harbor subset of genetic mutations that may result in distinct molecular characteristics, thus giving rise to the possibility of predictive biomarkers for potential therapies. Interestingly, in the realm of cancer genomics, the term “genetic mutations” is not just limited to the primary tumor/cancer. Rather, “genetic mutations” encompass gene mutations that may differ between the primary and relapse cancers, primary versus metastatic cancers, as well as therapy-induced genetic mutations in cancer patients. All of these factors may contribute to the patients' resistance to anticancer treatments and/or their relapse [56]. Identifying such genetic mutations may result in identification of predictive immune molecular signatures or immune biomarkers (cancer-specific neoantigens) among individual cancer patients which is a key step in developing patient-specific immunotherapies [55]. Interestingly, by evaluating specific genetic mutations the patient-specific cancer causing determinants can be identified and appropriately treated.

To date, several immunotherapies have been developed to treat cancers. While some of the treatments are already on the market or have been approved for clinical phase trials, through the use of high-throughput sequencing technologies, multiple genetic mutations can be identified to develop personalized treatments [56], thereby highlighting the importance of exploiting single-agent versus combination therapies in personalized cancer treatment plans [56].

The subsequent section in this review focuses on available immunotherapies for various cancers. However, it is important to note that through precision medicine efficacy of immunotherapies and cancer-related clinical outcomes can be improved by identifying and targeting patient-specific tumor antigens [55, 56].

## 8. Immunotherapies

Various cancers have unique triggers that result in their escape from the immune response making them more resistant to immunity [4]. Therefore, in efforts to treat various forms of cancers, scientific advances have been made towards developing therapies that exploit the immune system [40]. The specific types of treatments that focus on utilizing innate and adaptive immunity in oncology are referred to as cancer immunotherapies [54]. Cancer immunotherapies can be classified into several different categories: vaccines, monoclonal antibodies, recombinant cytokines, small molecules, and autologous T cells [1]. Depending on the location, cancer type, and the stage that the specific cancer is in, the type of therapy that is best suited for the patient is dictated.

US Food and Drug Administration (FDA) has approved Provenge in April 2010 as a therapeutic cancer vaccination for advanced prostate cancer [57]. It is a form of autologous cellular immunotherapy that consists of peripheral blood mononuclear cells, cytokine granulocyte macrophage colony stimulating factor (GM-CSF), and immunosurveillance of the tumor antigen-prostatic acid phosphatase (PAP) [54, 57]. The mechanism of action involves the uptake of PAP by APCs which is presented to T cells for activation, differentiation, and initiation of their effector functions [57]. GM-CSF is used to help stimulate the growth of APCs such as macrophages [58]. Some cancers, such as cervical cancers, arise from oncoviruses like human papilloma virus (HPV); therefore vaccinations against these oncoviruses can be incorrectly classified as vaccines against cancer [59].

**Figure 3:**
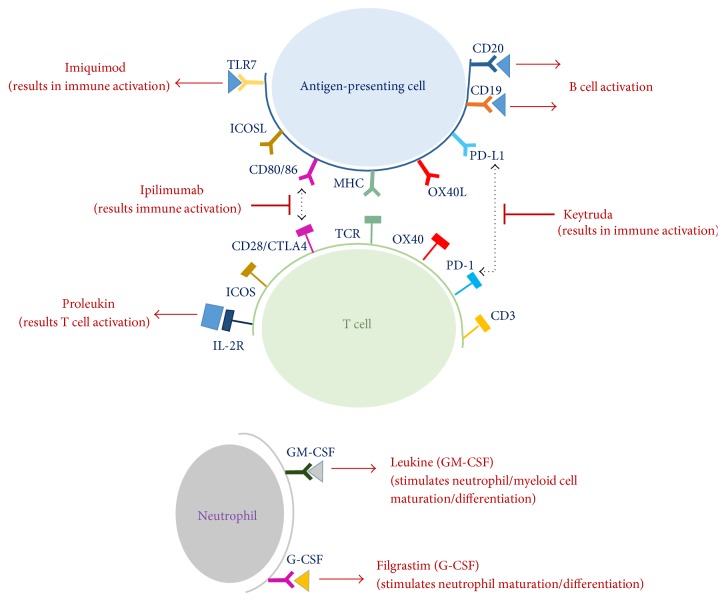
Immunotherapies in cancer. Several FDA approved immunotherapies have been approved in the market to prevent or control cancer progression [1]. On APCs (such as B cells), monoclonal antibodies targeting CD20 (Rituximab) results in downregulation of B cell activity [62]. Other antigen-presenting cells (macrophages and dendritic cells) are also activated by Imiquimod, a small molecule agonist for TLR7 [1]. For activation of T cells Keytruda (monoclonal antibody targeting PD-L1- PD-1 interaction) and Ipilimumab (antibody against CTLA4) are approved and in clinical use [1, 39]. Recombinant cytokines such as Proleukin (IL-2), Leukine (GM-CSF), and Filgrastim (G-CSF) are approved for stimulation/activation of T cells, myeloid cells, and neutrophils, respectively [1, 67].

Monoclonal antibodies are also used as cancer immunotherapies [60]. In March 2011, Ipilimumab was FDA approved for treatment of metastatic melanoma (malignant skin cancer). It is a monoclonal antibody targeting CTLA4 on T cells [54]; thereby it inhibits the suppressive effects of CTLA4 on T cells and allows activation of T cells for immune responses against specific cancers (Figure 3) [54]. Notably, Ipilimumab is also known to inhibit the immunosuppressive function of Tregs [54]. Similarly, IgG4 monoclonal antibody against PD-1 (Keytruda) has been on the market for treatment of melanoma patients (Figure 3) [61]. PD-1 is expressed on T cells and plays a role in immune-suppression by repressing T cell activation. However treatment with Keytruda prevents the inhibitory effects of PD-1 on T cells, thereby, allowing activation of T cells and immune responses against melanoma [61]. Similarly, Nivolumab is an FDA approved IgG4 monoclonal antibody that targets anti-PD1 in melanoma and squamous non-small-cell lung cancer patients [61]. It functions in the same manner as Keytruda [61]. Both of these anti-PD1 immunotherapies can also facilitate ADCC and result in cancer cell death [39]. In recent years, biotech companies such as Genentech have made efforts in developing potential monoclonal antibodies against the ligand for PD-1, PD-L1, as another mechanism to activate T cell-mediated immune responses and inhibit the immune-suppressive mechanisms of PD-1 in certain cancers [39]. There are also monoclonal antibodies conjugated to chemotherapy drugs or radioactive particles [62]. Zevalin® is a Yttirum-90 radiolabeled monoclonal antibody consisting of Rituximab which targets CD20 for activation of B cells and is used in treatments for non-Hodgkin's Lymphoma (Figure 3) [62]. Interestingly, monoclonal antibody immunotherapy targeting two different proteins simultaneously has also been developed and approved by FDA. This drug is known as Blincyto and it is a monoclonal antibody where one part attaches to CD19 on B cells and the other part of the antibody attaches to CD3 on T cells for T cell activation [63]. The CD19 cell surface marker assembles into a complex with other markers such as CD81 and CD21 (complement receptor) to lower the threshold for B cell activation [64]. In this manner, the normal T cells can recognize and mediate cytotoxicity on the cancerous B cells in efforts to eradicate them [63]. Immunotherapies utilizing monoclonal antibodies that target specific immune modulators or tumor-specific antigens help exploit the individual's own immune system to treat certain cancers.

Another immunotherapeutic approach for cancer treatments involves the use of recombinant cytokines or small molecules. For example, Proleukin is an FDA approved recombinant IL-2 cytokine for treatment of renal cancer and melanoma patients (Figure 3) [54]. This mechanism of action centers around the ability of IL-2 to promote T cell activation and the activation of other immune cells (lymphocytes) that express IL-2 receptors [47, 65]. Through Proleukin treatment, the immune system is activated and helps in controlling/killing the cancer cells [54]. A member of the IFN cytokine family, IFN-*α*2b, and pegylated IFN-*α*2b (Sylatron) were approved by FDA for treatment as adjuvant therapy in resected melanoma patients [66]. Sylatron is comprised of IFN-*α*2b conjugated to polyethylene glycol [66]. The polyethylene glycol reduces the immunogenicity of IFN-*α*2b by concealing it from the immune system until it reaches its target [66]. IFN-*α* cytokine is reported to be anti-inflammatory in cancer by repressing proliferation of cells, through induction of tumor suppressor genes and downregulation of oncogenes, as well as by upregulating MHC class I expression for immune responses [40]. Recombinant G-CSF, known as Filgrastim, has also been approved and been on the market to treat neutropenia in patients with certain forms of leukemia (Figure 3) [67]. Recombinant human G-CSF can bind to its corresponding receptors on neutrophil progenitor cells to stimulate neutrophil differentiation and maturation [67]. Increases in neutrophil production can help in cancer treatments by mediating cytotoxic effects on cancer cells, phagocytosing cancer cells, and by secreting cytokines that recruit other immune cells to the site of inflammation [2, 21]. However, as previously mentioned, neutrophils have dual functions in cancer pathogenesis and can have a role in cancer metastasis [42]. Therefore, Filgrastim should be used in combination with other anticancer immunotherapies [67]. Leukine, recombinant GM-CSF, is approved by the FDA (Figure 3) and functions in similar manner to Filgrastim. However, due to this also functioning as a macrophage colony stimulating factor, it helps increase myeloid cells (any leukocyte that is not B or T cells) in leukemic patients, as well as in individuals with bone marrow transplant [67].

The use of small molecules in cancer treatment as immunotherapies has also been increasing [1]. Plerixafor is a small molecule antagonist that inhibits the binding interaction of stromal cell-derived factor-1 (SDF-1) to the chemokine receptor, CXCR4 [1, 68]. This can prevent cancer metastasis and improve mobilization of hematopoietic stem cells in cancer patients, particularly pancreatic ductal adenocarcinoma patents [1]. Interaction of SDF-1 with CXCR4 mediates functions such as attracting lymphocytes in certain conditions and having critical functions for homing of hematopoietic stem cells to the bone marrow [69]. For treatment of basal cell carcinoma, Imiquimod, a small molecule agonist for TLR7 on dendritic cells and macrophages, is being used (Figure 3) [1]. Imiquimod-mediated TLR7 activation induces secretion of proinflammatory cytokines, suppresses Tregs, and induces Th1 cell-mediated activation of NK cells to eradicate cancer cells [1].

A novel cancer immunotherapy involves the use of a cancer patient's own immune cells (such as T cells) which are collected, genetically altered, proliferated to increase the number of cells, and transferred back to the patient [54]. This innovative form of cancer immunotherapy is known as adoptive T cell transfer. The alteration (via retroviral gene transfer) of autologous T cells prior to being transferred back into the patient comes from the concept of chimeric antigen receptors (CARs) on T cells (CAR-T cells) [70]. CAR-T cells are comprised of variable regions for identifying various antigens and have various downstream (intracellular) T cell signaling components [54]. While no FDA approved CAR-T cell immunotherapy is on the market just yet, clinical trials have been testing the CAR-T cell therapy where CAR is recognizing CD19 on B cells in B cell lymphoma patients [54]. Other common treatments for cancer patients involve the use of chemotherapy and/or radiation therapy [71]. Chemotherapy utilizes agents that are cytotoxic to rapidly proliferating cancer cells but depending on the regimen used can also be toxic to hematopoietic cells in the bone marrow [71], whereas radiation therapy exploits ionizing radiation to target cancer cell death by damaging their DNA. However, this also damages the adjacent normal healthy cells [71]. In hematological cancer malignancies such as leukemia or damage done to the bone marrow as a result of chemotherapy/radiation therapy, bone marrow transplants are also commonly performed [72]. While this section has focused on different forms of cancer immunotherapies as an individual therapy, the reality is that these cancer immunotherapies may work more effectively when used in combination [39]. Currently, cancer patients are being treated by therapy regimens that include chemotherapy/radiation therapy along with targeted drugs that can affect various factors in cancer progression such as immune responses, DNA damage, or growth factors [73]. Interestingly, there also has been interest in trying to combine immunotherapies [39], due to the concept of cancers “escaping” the immune responses during the immune-editing process [74]. Wolchok's group conducted a phase I trial in which Ipilimumab (anti-CTLA4) plus Nivolumab (anti-PD1) immunotherapies were used together for advanced melanoma patients [39]. Ipilimumab promotes activation and priming of T cells and Nivolumab prevents interaction of PD-L1 with PD-1 on the cancer cells. Through these studies, Wolchok and colleagues confirmed that Ipilimumab's targeted effect, efficacy, and outcome are better when simultaneously given with Nivolumab [75]. These studies have provided the foundation for assessing other potential combinations of immunotherapies in adjunct with chemotherapy/radiation therapy to potentially improve survival outcomes for cancer patients.

## 9. Future Perspectives/Conclusions

The active role of the immune system in oncogenesis has been appreciated for over a century [4]. However, due to cancer heterogeneity and the distinct immune responses that can be activated, the field of immune-oncology is constantly evolving. In the past few decades, several scientific advancements have been made that increase our understanding of various immune mechanisms that contribute to cancer pathogenesis. In addition, the identification of immune biomarkers could potentially be exploited for cancer immunotherapies [5]. While several forms of immunotherapies have been approved by the FDA or have entered the early phases of the clinical trials, there are other forms of immunotherapies that still merely remain a concept. Research involving the immunooncology paradigm has directed its efforts towards translating some of these conceptual immunotherapies into treatments for patients. For instance, in addition to CTLA4 and PD-1, other antagonistic antibodies can be developed to target other immunomodulatory receptors on T cell surface that are inhibitory to its function such as antibodies against LAG-3, VISTA, and BTLA [54]. All three of these receptors are repressive to T cells functions; therefore, development of antibodies that target and block immunosuppressive receptors on T cells will lead to optimal T cell activation and immune responses against cancer [54]. In contrast, T cells also have activating receptors such as CD28, OX40, and GITR that can be targeted with agonistic antibodies to promote T cell activation [54]. Notably, interest in small molecule inhibitors to pharmacologically intervene and block immunosuppression is emerging as another driving force in cancer immunotherapies [1]. Currently, phase II clinical trials are being performed using a small inhibitor against IDO for metastatic melanoma [1]. IDO is heme-containing dioxygenase that is expressed by APCs and is upregulated in tumors/cancer cells [1]. It mediates the conversion of tryptophan to kynurenine [1] and plays a role in immunosuppression during cancer pathogenesis [1], by inducing differentiation of Tregs [1]. Thus, by inhibiting IDO, Tregs are not induced, resulting in activation of immune responses [1]. Small molecule inhibitors targeting chemokine receptors or adenosine pathways are also being investigated for cancer immunotherapies due to their ability to recruit or activate tissue-associated macrophages (TAMs), respectively [1]. These TAMs can promote cancer or tumor progression via angiogenesis and neovasculature formation [1].

Despite the progresses made in this field, one of the biggest challenges that still remains is understanding how immunooncology and cancer immunotherapies differ between adults and children [19]. While research involving the role for the immune system in pediatric cancer has been on the rise, drug development and cancer immunotherapies for children diagnosed with cancer significantly lags behind when compared to adult cancer treatments [19]. Due to the variability associated with the type of cancer, the cancer location, and the somewhat different composition of the immune cells in adults versus children, it becomes imperative to elucidate what immune cell differences are so that the proper cancer immunotherapies are selected to target specific patients [19]. Similarly, it is also becoming increasingly evident that even if two individuals of the same age group (adults or children) are diagnosed with the same form of cancer, the type of immune responses controlling/promoting the cancer and the type of immunotherapies to treat the cancer may still vary among these two individuals. This variability in cancer pathogenesis and treatment between individuals can be attributed to factors such as genetic polymorphisms which can differentially regulate the immunoediting process involved in cancer pathogenesis, as well as differentially regulating the efficacy of various cancer immunotherapies [60, 76]. Consequently, the next frontier in immunooncology will be focused on developing personalized cancer immunotherapies tailored to each individual patient.

## References

[1] Adams J. L., Smothers J., Srinivasan R., Hoos A. (2015). Big opportunities for small molecules in immuno-oncology. *Nature Reviews Drug Discovery*.

[2] Murphy K., Travers P., Walport M. (2008). Principles of innate and adaptive immunity. *Janeway's Immunobiology*.

[3] Vesely M. D., Kershaw M. H., Schreiber R. D., Smyth M. J. (2011). Natural innate and adaptive immunity to cancer. *Annual Review of Immunology*.

[4] Schreiber R. D., Old L. J., Smyth M. J. (2011). Cancer immunoediting: integrating immunity's roles in cancer suppression and promotion. *Science*.

[5] Hoos A., Britten C. M. (2012). The immuno-oncology framework enabling a new era of cancer therapy a new era of cancer therapy. *OncoImmunology*.

[6] Siegel R. L., Miller K. D., Jemal A. (2015). Cancer statistics, 2015. *CA: A Cancer Journal for Clinicians*.

[7] Pinho S. S., Reis C. A. (2015). Glycosylation in cancer: mechanisms and clinical implications. *Nature Reviews Cancer*.

[8] Sharma S., Kelly T. K., Jones P. A. (2009). Epigenetics in cancer. *Carcinogenesis*.

[9] Akhavan-Niaki H., Samadani A. A. (2013). DNA methylation and cancer development: molecular mechanism. *Cell Biochemistry and Biophysics*.

[10] Ehrlich M. (2002). DNA methylation in cancer: too much, but also too little. *Oncogene*.

[11] Lodish H., Berk A., Zipursky S. L., Matsudaira P., Baltimore D., Darnell J. (2000). Tumor cells and the onset of cancer. *Molecular Cell Biology*.

[12] Artac M., Bozcuk H., Ozdogan M. (2005). Different clinical features of primary and secondary tumors in patients with multiple malignancies. *Tumori*.

[13] Reymond N., D'Água B. B., Ridley A. J. (2013). Crossing the endothelial barrier during metastasis. *Nature Reviews Cancer*.

[14] Rundhaug J. E. (2003). Matrix metalloproteinases, angiogenesis, and cancer: commentary re: A. C. Lockhart et al., Reduction of wound angiogenesis in patients treated with BMS-275291, a broad spectrum matrix metalloproteinase inhibitor. *Clinical Cancer Research*.

[15] Nishida N., Yano H., Nishida T., Kamura T., Kojiro M. (2006). Angiogenesis in cancer. *Vascular Health and Risk Management*.

[16] Blanpain C. (2013). Tracing the cellular origin of cancer. *Nature Cell Biology*.

[17] Sehn L. H. (2015). Introduction to a clinical review series on aggressive B-cell lymphoma. *Blood*.

[18] Fermand J. P., James J. M., Herait P., Brouet J. C. (1985). Association chronic lymphocytic leukemia and multiple myeloma: origin from a single clone. *Blood*.

[19] Boklan J. (2006). Little patients, losing patience: pediatric cancer drug development. *Molecular Cancer Therapeutics*.

[20] Hanahan D., Weinberg R. A. (2011). Hallmarks of cancer: the next generation. *Cell*.

[21] Murphy K., Travers P., Walport M. (2008). Pattern recognition in the innate immune system. *Janeway's Immunobiology*.

[22] Dominguez-Villar M., Gautron A.-S., de Marcken M., Keller M. J., Hafler D. A. (2015). TLR7 induces anergy in human CD4^+^ T cells. *Nature Immunology*.

[23] Dinarello C. A. (2007). Historical review of cytokines. *European Journal of Immunology*.

[24] Wills-Karp M. (2007). Complement activation pathways: a bridge between innate and adaptive immune responses in asthma. *Proceedings of the American Thoracic Society*.

[25] Sun J. C., Lanier L. L. (2009). Natural killer cells remember: an evolutionary bridge between innate and adaptive immunity?. *European Journal of Immunology*.

[26] Murphy K., Travers P., Walport M. (2008). Structural variation in immunoglobulin constant regions. *Janeway's Immunobiology*.

[27] Chien Y.-H., Jores R., Crowley M. P. (1996). Recognition by *γ*/*δ* T cells. *Annual Review of Immunology*.

[28] Koretzky G. A. (2010). Multiple roles of CD4 and CD8 in T cell activation. *Journal of Immunology*.

[29] Luckheeram R. V., Zhou R., Verma A. D., Xia B. (2012). CD4 +T cells: differentiation and functions. *Clinical and Developmental Immunology*.

[30] Podojil J. R., Miller S. D. (2009). Molecular mechanisms of T-cell receptor and costimulatory molecule ligation/blockade in autoimmune disease therapy. *Immunological Reviews*.

[31] Janeway C. A., Travers P., Walport M. (2001). B-cell activation by armed helper T cells. *Immunobiology: The Immune System in Health and Disease*.

[32] Schroeder H. W., Cavacini L. (2010). Structure and function of immunoglobulins. *Journal of Allergy and Clinical Immunology*.

[33] Terabe M., Berzofsky J. A. (2008). The role of NKT cells in tumor immunity. *Advances in Cancer Research*.

[34] Chaplin D. D. (2010). Overview of the immune response. *Journal of Allergy and Clinical Immunology*.

[35] Dunkelberger J. R., Song W.-C. (2010). Complement and its role in innate and adaptive immune responses. *Cell Research*.

[36] Kwan W.-H., Van Der Touw W., Heeger P. S. (2012). Complement regulation of T cell immunity. *Immunologic Research*.

[37] Waldhauer I., Steinle A. (2008). NK cells and cancer immunosurveillance. *Oncogene*.

[38] Warrington R., Watson W., Kim H., Antonetti F. (2011). An introduction to immunology and immunopathology. *Allergy, Asthma & Clinical Immunology*.

[39] Chen D. S., Mellman I. (2013). Oncology meets immunology: the cancer-immunity cycle. *Immunity*.

[40] Dunn G. P., Koebel C. M., Schreiber R. D. (2006). Interferons, immunity and cancer immunoediting. *Nature Reviews Immunology*.

[41] Pio R., Corrales L., Lambris J. D. (2014). The role of complement in tumor growth. *Advances in Experimental Medicine and Biology*.

[42] Gregory A. D., Houghton A. M. (2011). Tumor-associated neutrophils: new targets for cancer therapy. *Cancer Research*.

[43] Palucka K., Banchereau J. (2012). Cancer immunotherapy via dendritic cells. *Nature Reviews Cancer*.

[44] Gogoi D., Chiplunkar S. V. (2013). Targeting gamma delta T cells for cancer immunotherapy: bench to bedside. *The Indian Journal of Medical Research*.

[45] Sharpe A. H. (2009). Mechanisms of costimulation. *Immunological Reviews*.

[46] Harris T. J., Drake C. G. (2013). Primer on tumor immunology and cancer immunotherapy. *Journal for Immunotherapy of Cancer*.

[47] Minami Y., Kono T., Miyazaki T., Taniguchi T. (1993). The IL-2 receptor complex: its structure, function, and target genes. *Annual Review of Immunology*.

[48] Crespo J., Sun H., Welling T. H., Tian Z., Zou W. (2013). T cell anergy, exhaustion, senescence, and stemness in the tumor microenvironment. *Current Opinion in Immunology*.

[49] Contardi E., Palmisano G. L., Tazzari P. L. (2005). CTLA-4 is constitutively expressed on tumor cells and can trigger apoptosis upon ligand interaction. *International Journal of Cancer*.

[50] Nishikawa H., Sakaguchi S. (2010). Regulatory T cells in tumor immunity. *International Journal of Cancer*.

[51] Gobert M., Treilleux I., Bendriss-Vermare N. (2009). Regulatory T cells recruited through CCL22/CCR4 are selectively activated in lymphoid infiltrates surrounding primary breast tumors and lead to an adverse clinical utcome. *Cancer Research*.

[52] Mittal D., Gubin M. M., Schreiber R. D., Smyth M. J. (2014). New insights into cancer immunoediting and its three component phases-elimination, equilibrium and escape. *Current Opinion in Immunology*.

[53] Dunn G. P., Old L. J., Schreiber R. D. (2004). The three Es of cancer immunoediting. *Annual Review of Immunology*.

[54] Mellman I., Coukos G., Dranoff G. (2011). Cancer immunotherapy comes of age. *Nature*.

[55] Mandal R., Chan T. A. (2016). Personalized oncology meets immunology: the path toward precision immunotherapy. *Cancer Discovery*.

[56] Bluestone J. A., Tang Q. (2015). Immunotherapy: making the case for precision medicine. *Science Translational Medicine*.

[57] Cheever M. A., Higano C. S. (2011). PROVENGE (sipuleucel-T) in prostate cancer: the first FDA-approved therapeutic cancer vaccine. *Clinical Cancer Research*.

[58] Shi Y., Liu C. H., Roberts A. I. (2006). Granulocyte-macrophage colony-stimulating factor (GM-CSF) and T-cell responses: what we do and don't know. *Cell Research*.

[59] Schiller J. T., Lowy D. R. (2010). Vaccines to prevent infections by oncoviruses. *Annual Review of Microbiology*.

[60] Weiner L. M., Dhodapkar M. V., Ferrone S. (2009). Monoclonal antibodies for cancer immunotherapy. *The Lancet*.

[61] Aris M., Barrio M. M. (2015). Combining immunotherapy with oncogene-targeted therapy: a new road for melanoma treatment. *Frontiers in Immunology*.

[62] Krasner C., Joyce R. M. (2001). Zevalin™: 90ytrium labeled anti-CD20 (Ibritumomab tiuxe tan), a new treatment for non-Hodgkin's lymphoma. *Current Pharmaceutical Biotechnology*.

[63] Przepiorka D., Ko C.-W., Deisseroth A. (2015). FDA approval: blinatumomab. *Clinical Cancer Research*.

[64] Wang K., Wei G., Liu D. (2012). CD19: a biomarker for B cell development, lymphoma diagnosis and therapy. *Experimental Hematology & Oncology*.

[65] Nelson B. H. (2004). IL-2, regulatory T cells, and tolerance. *The Journal of Immunology*.

[66] Patel J. N., Walko C. M. (2012). Sylatron: a pegylated interferon for use in melanoma. *The Annals of Pharmacotherapy*.

[67] Buchsel P. C., Forgey A., Grape F. B., Hamann S. S. (2002). Granulocyte macrophage colony-stimulating factor: current practice and novel approaches. *Clinical Journal of Oncology Nursing*.

[68] Uy G. L., Rettig M. P., Cashen A. F. (2008). Plerixafor, a CXCR4 antagonist for the mobilization of hematopoietic stem cells. *Expert Opinion on Biological Therapy*.

[69] Tögel F., Isaac J., Hu Z., Weiss K., Westenfelder C. (2005). Renal SDF-1 signals mobilization and homing of CXCR4-positive cells to the kidney after ischemic injury. *Kidney International*.

[70] Magee M. S., Snook A. (2014). Challenges to chimeric antigen receptor (CAR)-T cell therapy for cancer. *Discovery Medicine*.

[71] Formenti S. C., Demaria S. (2009). Systemic effects of local radiotherapy. *The Lancet Oncology*.

[72] Duran-Struuck R., Dysko R. C. (2009). Principles of Bone Marrow Transplantation (BMT): providing optimal veterinary and husbandry care to irradiated mice in BMT studies. *Journal of the American Association for Laboratory Animal Science*.

[73] Li F., Zhao C., Wang L. (2014). Molecular-targeted agents combination therapy for cancer: developments and potentials. *International Journal of Cancer*.

[74] Drake C. G. (2012). Combination immunotherapy approaches. *Annals of Oncology*.

[75] Wolchok J. D., Kluger H., Callahan M. K. (2013). Nivolumab plus Ipilimumab in advanced melanoma. *The New England Journal of Medicine*.

[76] Dong L. M., Potter J. D., White E., Ulrich C. M., Cardon L. R., Peters U. (2008). Genetic susceptibility to cancer: the role of polymorphisms in candidate genes. *Journal of the American Medical Association*.

